# Investigation of target genes and potential mechanisms related to compound Xiao-ai-fei honey ointment based on network pharmacology and bioinformatics analysis

**DOI:** 10.1097/MD.0000000000034629

**Published:** 2023-08-11

**Authors:** Kayisaier Abudurousuli, Ziruo Talihati, Sendaer Hailati, Meng Yuan Han, Muhadaisi Nuer, Nawaz Khan, Nulibiya Maihemuti, Dilihuma Dilimulati, Nuerbiye Nueraihemaiti, Jimilihan Simayi, Wenting Zhou

**Affiliations:** a Department of Pharmacology, School of Pharmacy, Xinjiang Medical University, Urumqi, P.R. China.

**Keywords:** bioinformatics analysis, gastric cancer, MMP2, MMP9, molecular docking, network pharmacology

## Abstract

Compound Xiao-ai-fei honey ointment (CXHO) is an anticancer preparation with a long history in Uyghur folk medicine in China and has been used for the treatment of gastric cancer (GC) in Xinjiang, China. Nevertheless, the mechanism of its anticancer effect remains to be investigated.

**Methods:**

Bioactive ingredients of CXHO were retrieved from the Traditional Chinese Medicine Systems Pharmacology Database and Analysis Platform database. Target genes of ingredients were acquired via the PubChem and Swiss target prediction database. Gene expression profiling of GC was obtained from GSE54129 in the GEO database and analyzed using the limma package in R. The hub genes associated with CXHO in GC were validated using the TIMER2.0 database, GEPIA2 database and Auto Dock tools. The effect of CXHO on migration of GC cells was detected by Transwell chamber assay and Wound healing assay. The effect of CXHO on expression levels of MMP2/MMP9 and NF-κb, PI3K/AKT signaling pathway was detected by Western blot assay.

**Results:**

Forty-five bioactive ingredients and their 819 related genes were found. A total of 462 differentially expressed genes were identified between GC patients and healthy controls. Seventeen common target genes were identified as hub genes CXHO against GC. Among them, MMP2 and MMP9 were significantly associated with tumor immune infiltrates and had good binding affinity with effective ingredients. Moreover, we validated the mRNA and protein expression levels and prognostic value of MMP2 and MMP9 by different databases. In addition, Kyoto encyclopedia of genes and genomes and gene ontology analyses showed that the 17 common target genes were mainly involved in steroid hormone biosynthesis and cancer-related pathways. Experimental results showed that CXHO inhibited migration of GC cells and down regulated the expression levels of MMP2/MMP9, NF-κb. In addition, CXHO can inhibited PI3K/AKT signaling pathway.

**Conclusion:**

We identified and experimental validated 2 pivotal target genes of CXHO against GC and preliminarily analyzed the potential mechanisms by which CXHO inhibits the development of GC. All these findings support CXHO as a promising drug for the treatment of GC.

## 1. Introduction

Gastric cancer (GC) is one of the most commonly diagnosed cancers and the fourth leading cause of cancer-related deaths worldwide according to Global Cancer Statistics 2020.^[1]^ The prognosis of patients with advanced GC is poor, with a median survival of less than 1 year.^[2]^ Although immunotherapy is the treatment of choice for specific subtypes of GC, the heterogeneity of GC remains a key obstacle to the development of effective drugs to improve the prognosis of GC patients.^[3]^ Therefore, there is an urgent need to develop new drugs to improve the survival of GC patients.

Compound Xiao-ai-fei honey ointment (CXHO) is an anticancer preparation with a long history in Uyghur folk medicine in China. It has been used for the treatment of GC and esophageal cancer in Xinjiang, China. Recent studies have shown that 1213 GC patients treated with CXHO have improved prognostic outcomes.^[4–6]^ According to the literature, CXHO is made of several bioactive components, including *Bungarus multicinctus, Piperis Longi Fructus, Rhizoma Alpiniae Officinarum, Piper nigrum L*, and *Zingiber officinale Roseco*.^[7–11]^

*B multicinctus* (Bm), the juvenile snake dried body of the Chinese krait,^[12]^ contains more than 20 elements,^[13]^ and has been used to treat rheumatic disease, tetanus and leprosy,^[14]^ due to its anti-inflammatory and analgesic effects.^[15]^ *Piperis Longi Fructus* (PLF), which is made from the mature and immature fruit of *Piper longum* L,^[16]^ has been widely used in traditional Chinese medicine (TCM) and Indian medicine. Studies have shown that components of PLF have anti-inflammatory, antitumor, and antidepressant effects.^[17]^ *Rhizoma Alpiniae Officinarum* (RAO), the dry root and rhizome of *Alpinia officinarum*,^[18]^ is reported to have antioxidant, antidiabetic, anti-inflammatory, and anticoagulation effects.^[19,20]^ *P nigrum* L. (PnL) is a pepper made of flowering vines of the Piperaceae family,^[21]^ and has a variety of pharmacological functions, including gastric protection and antioxidation activity.^[22,23]^ Recent studies^[24–26]^ have found that piperine has shown inhibitory effects on multiple types of cancer, including pancreatic cancer, breast cancer, and GC. *Zingiber officinale Roseco* (ZoR) is the dried root of ginger family plants and has been commonly used in clinical practice to relieve vomiting and enhance gastric function.^[27]^ Nevertheless, the potential mechanisms of the antitumor effects of these drugs remain to be investigated.

To this end, we applied a combination bioinformatics analysis to explore the potential pharmacological mechanisms and investigate the bioactive targets and pathways of CXHO. The findings of the present study are expected to contribute to the design of therapeutic strategies for GC.

## 2. Materials and methods

### 2.1. Ethical approval

Ethical approval was not necessary because this study did not involve animals or human subjects (tissues).

### 2.2. Data acquisition and preprocessing

Gene expression profiling of 111 GC samples and 21 healthy controls was retrieved from GSE54129 in the GEO database. Samples in GSE54129 were all profiled based on the sequencing platform of GPL570 (Affymetrix Human Genome U133 Plus 2.0 Array). Data normalization and probe summarization were performed by robust multichip analysis (RMA) using R Bioconductor package.

### 2.3. Identification of active compounds and putative target proteins of CXHO

The active compounds of CXHO and their putative target proteins were obtained from the Traditional Chinese Medicine Systems Pharmacology (TCMSP) Database and Analysis Platform (https://old.tcmsp-e.com/tcmsp.php). As a unique database containing a large number of herbs, active ingredients, and their targets, TCMSP contains pharmacokinetic properties of active compounds, such as oral bioavailability (OB), drug-likeness (DL) and so on. OB refers to the extent and rate at which the oral drug enters the blood circulatory system.^[28]^ The greater the OB value of a compound, the more likely it is to become an effective drug. DL properties refers to the comparability of structural features and physicochemical properties of an ingredient to a licensed drug candidate.^[29]^ Due to poor pharmacological activity, most compounds in TCM cannot become effective drugs. OB ≥ 30% and DL ≥ 0.18 are considered as the criteria for screening clinical drugs. The putative target proteins of active compounds were collected from TCMSP database, because the database contains the target proteins of each active compound. In addition, we also utilized Swiss Target Prediction database (http://www.swisstargetprediction.ch/) to predict target proteins of active ingredients based on probability ≥ .1. The common genes obtained by overlapping these target proteins from TCMSP and Swiss Target Prediction databases were considered the putative target proteins of CXHO.

### 2.4. Prediction of the GC related targets of CXHO

GC related targets collected from Gene Cards database(https://genecards.org/) based on score > 10. Using Venn diagram screen common genes both CXHO and GC.

### 2.5. Identification of differentially expressed genes

Differentially expressed genes (DEGs) between GC tissue and normal control tissue were analyzed using the limma package in R. This R software package is designed for the analysis of gene expression of microarray and RNA-seq data. The core algorithm employs a generalized linear model, log-normal distribution, trimmed mean of M-values, and F tests. The selection thresholds for DEGs in this study included *P* < .05 and |log_2_FC| > 2.

### 2.6. Construction of protein–protein interaction network

A protein–protein interaction (PPI) network is a computational approach to interrogate the interactions between multiple proteins.^[30]^ We constructed a PPI network of 462 DEGs using the STRING database (https://string-db.org), reanalyzed the PPI network using the Cytohubba plugin in Cytoscape to obtain the hub genes.

### 2.7. Functional enrichment analysis

We utilized 2 canonical database resources to investigate the biological function of genes of interest, i.e., the gene ontology (GO) database and Kyoto encyclopedia of genes and genomes (KEGG). GO consists of 3 parts: biological processes, cellular components, and molecular functions.^[31,32]^ KEGG is a database for understanding molecular functions and biological systems and represents one of the most commonly used bioinformatics databases.^[33]^ Sanger Box 3.0 online platform (http://sangerbox.com/) was used to perform KEGG and GO analyses.

### 2.8. Bioinformatics analyses of target genes

Bioinformatics analyses of target genes mainly included 3 aspects: survival analysis, gene expression analysis, and correlation analysis. The Kaplan–Meier database (https://kmplot.com/analysis) was used for survival analysis of relevant genes. The TIMER2.0 database(http://timer.comp-genomics.org) was exploited to perform the pan-cancer analysis of related genes. GEPIA2 database (https://GEPIA2.cancer-pku.cn) was utilized to verify the expression levels of interested genes. The Human Protein Atlas database (http://timer.proteinatlas.org) was employed to validate the protein levels of target genes.

### 2.9. Molecular docking

Molecular docking is regarded as a key technique for structure-based research and drug development.^[34]^ Here, molecular docking was used to further investigate the interaction between hub genes and the active ingredients β-sitosterol (SIT), isorhamnetin (IH), and medicarpin. First, the mol2 format of these bioactive ingredients was collected from the PubChem database and converted to the pdbqt format using PyMOL and Open Babel software. The structure of the potential receptors was obtained from the RCSB Protein Data Bank (RCSB PDB; https://www.wwpdb.org). Water molecules and ligands were removed by PyMOL software. Molecular docking was performed by Auto Dock, a suite of automated docking tools that are designed to predict how small substrates or drug candidates bind to a receptor of known 3D structure. In the present study, Auto Dock was employed to exploit the binding pattern and affinity between the active compounds and the macromolecules. All parameters were set in default. The findings were visualized using PyMOL software and Ligplus software.

### 2.10. Experimental validation

#### 2.10.1. Preparation of CXHO.

Compound Xiao Ai Fei Honey ointment was prescribed by Xinjiang Uyghur Autonomous Region Uyghur Medical Hospital. Freeze-dried the CXHO. Dissolved into 500 mg/mL with DMSO, centrifuged at 10,000 rpm for 30 minutes, collected the supernatant, and filtered with a 0.22 μm filter for later use. When used, diluted to the desired concentration with a complete medium and prepare ready-to-use.

#### 2.10.2. Cell culture.

Human AGS and MNK-45 cells were selected for the following experiments. AGS and MNK-45 cells were purchased from Procell Biotech Co., Ltd. (Procell, Wuhan, China); Cells were cultured in RPIM1640 medium supplemented with 10% FBS, 100 U/mL penicillin, and 100 mg/mL streptomycin and kept at 37 °C in a humidified chamber with 5% CO_2_.

#### 2.10.3. Wound healing and Transwell assay.

For wound healing assays, AGS cells were incubated in 6-well plates that were 100% confluent. Used a plastic pipette tip to scrape the exposed area on the cell monolayer. Removed the medium and washed the monolayer 3 times with PBS. Then, medium containing different concentrations of CXHO (50 μg/mL, 250 μg/mL, and 500 μg/mL) was added to each well, and cell movements into the wound area were obtained after 0, 12, 24,48 hours and incubated under a microscope for 24 and 48 hours. For the transwell chamber assay, AGS cells (1 × 10^4^ cells/well) were seeded into an incubator filled with 300 μL serum-free 1640 and then incubated at 37° with or without CXHO for 24 and 48 hours. Invading cells were fixed with 4% paraformaldehyde for 30 minutes, stained with crystal violet solution for 30 minutes, and then counted with photographs with a light microscope.

#### 2.10.4. Western blotting.

For cell samples, MNK-45 cells and AGS cells (5 × 10^5^ cells/well) were seeded in 6-well plates. After incubation overnight, cells were treated with or without CXHO for 24 hours. Pancreatic digestion collects cells. The expression levels of MMP2, MMP9, PI3K, and P-AKT were detected by western blotting. Briefly, whole cell extracts lysed with RIPA buffer supplemented with proteinase inhibitors (1% PMSF, 0.5% aprotinin, and 0.5% leupeptin) and phosphatase inhibitors (1 mM Na3VO4) in ice lysis for 30 minutes. Subsequently, the lysates were centrifuged at 4 ºC at 10,000 rpm for 15 minutes. Used of bovine serum albumins (BSA; Solarbio, Beijing, China) as standard to detect protein concentration. Equal amounts of protein in each sample were separated by sodium dodecyl sulfate-polyacrylamide gel electrophoresis (SDS-PAGE) and transferred to a polyvinylidene fluoride membrane (PVDF; Biorad, Hercules, CA). Next, the membrane was blocked with 5% BSA in tris-buffered saline-Tween 20 (TBST) buffer (10 mmol/L tris, 150 mmol/L NaCl, 1% Tween 20, pH 7.4) for 2 hours at room temperature. The blots were incubated overnight at 4 ºC with a primary antibody (anti-PI3K, anti-PAKT, anti-MMP2, anti-MMP9 at 1:1000; Affinity, San Francisco, CA). After 3 washes with tris-buffered saline-Tween 20 buffer, incubated the blotting with the secondary antibody (Affinity, San Francisco, CA) for 2 hours at room temperature. Immunoreactivity was determined using an advanced ECL kit (Thermofisher, Waltham, MA) and visualized using a chemiluminescence imaging system (Biorad).

#### 2.10.5. Statistical analysis.

Statistical analysis using Prism 9.1.5 software. Data were expressed as mean ± standard deviation and analyzed using the One-way ANNOVA test. If *P* < a value of .05, the between-group difference was considered statistically significant.

## 3. Results

### 3.1. Overview of the present study

**Figure 1.**
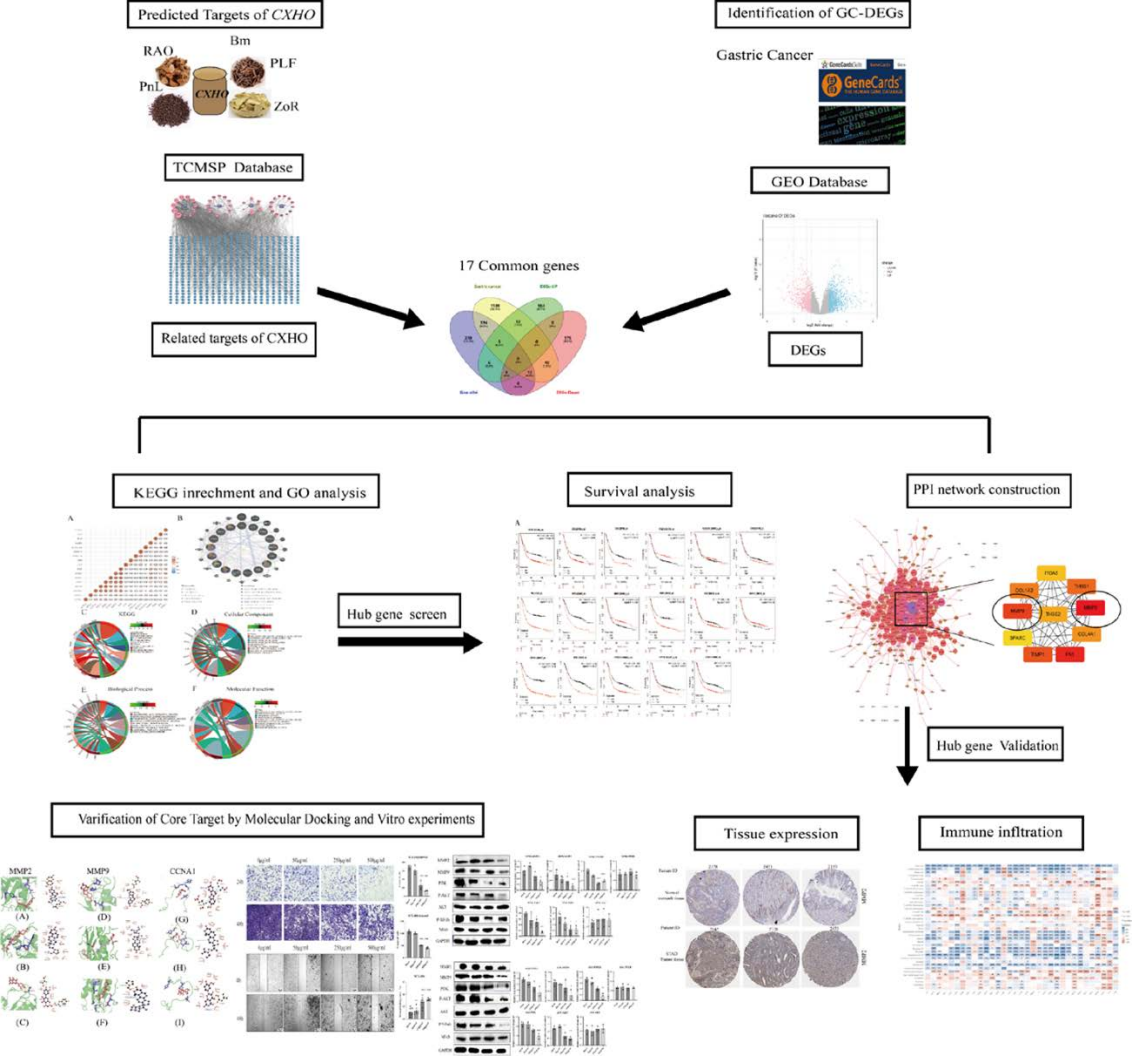
Workflow diagram of the network and bioinformatics study.

The present study focused on identifying the target genes of CXHO and investigating the potential molecular mechanisms of its function. The pipeline of the current research is shown in Figure 1. First, we identified active compounds and targets of CXHO using the TCMSP database and determined the DEGs between GC patients and healthy controls based on gene expression data from the GEO database using the limma algorithm. Then, we intersected the gene list generated from the TCMSP database with DEGs between GC patients and healthy controls to obtain potential hub genes associated with CXHO. The online tool STRING and the JAVA software Cytoscape were utilized to identify 2 hub genes: MMP2 and MMP9. Afterward, we validated the prognostic outcomes and the mRNA and protein levels of the 2 hub genes using 3 different online tools. Eventually, molecular docking was used to further investigate the interaction between hub genes and the active ingredients SIT, IH, and medicarpin.

### 3.2. Acquisition of active compounds and targets of the CXHO

**Figure 2.**
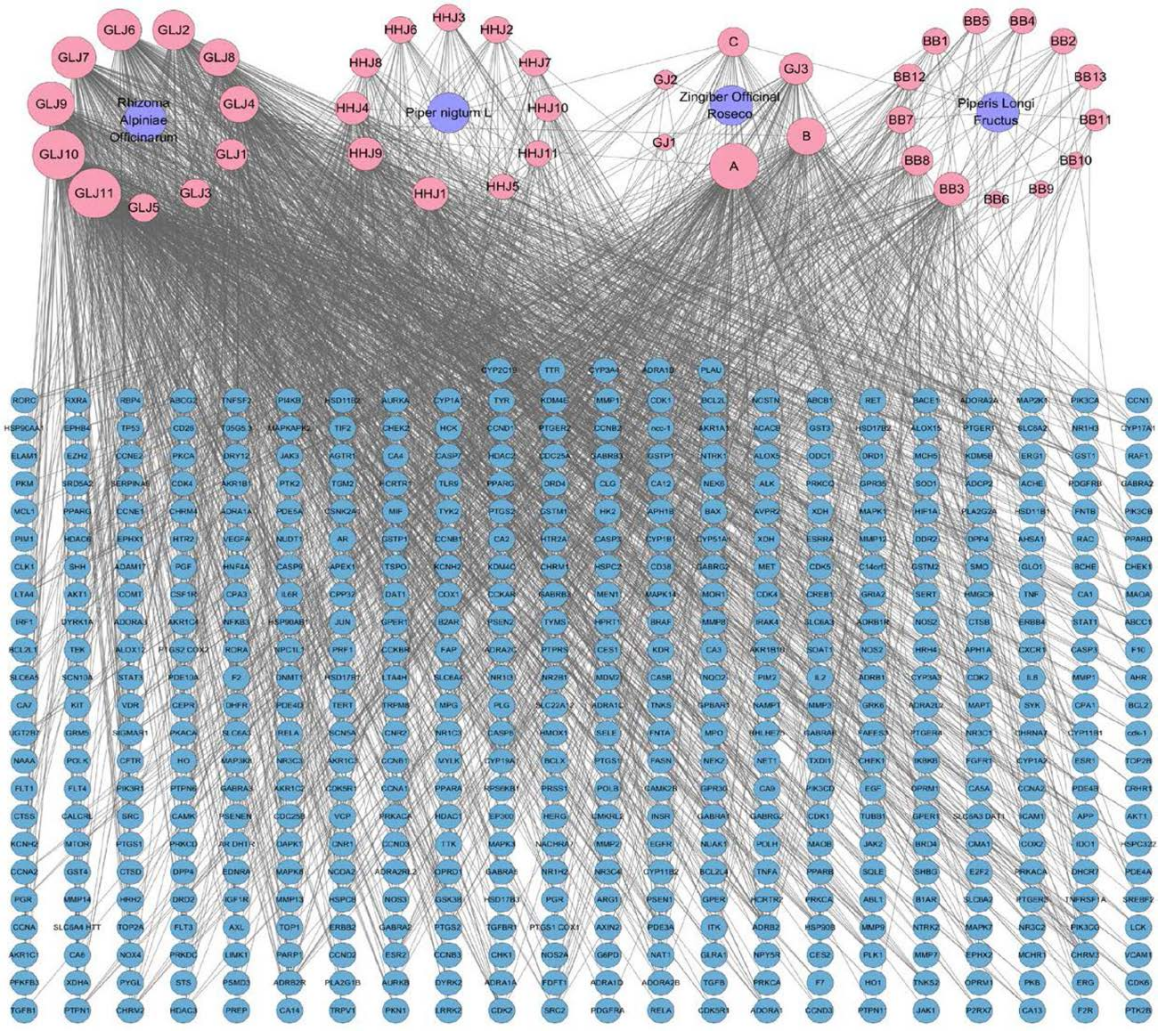
CXHO Compound-Target (C-T) network diagram; pink circle represents the drug, and active ingredient, and blue circle represent the target.

First, we identified that CXHO contains 4 flavor herbs, *Piperis Longi Fructus* (PLF), *Rhizoma Alpiniae Officinarum* (RAO), *P nigrum L* (PnF), and *Zingiber officinale Roseco* (ZoR), based on the TCMSP database (OB ≥ 30%, DL ≥ 0.18). Specifically, PLF has 13 compounds, among which 4 ingredients are unique to PLF; RAO has 14 compounds, among which 9 ingredients are unique to RAO; PnL has 12 ingredients, among which 8 ingredients are unique to PnL; and ZoR has 5 active ingredients. For further investigation, we excluded 10 active ingredients without corresponding target information, including MOL002501, MOL000483, MOL002866, MOL002864, MOL002863, MOL001616, MOL001607, MOL001588, MOL001555, and MOL002556. In addition, SIT and sitosterol were found to be the common active constituents of PLF and ZoR; N-isobutyl-2,4-icosadienamide was found to be the common active ingredient of RAO and PnL. After identification of the active ingredients in CXHO, we imported these active ingredient names into the PubChem database to obtain canonical Smiles and then imported canonical Smiles to the Swiss Target Prediction database to obtain the corresponding targets of the bioactive ingredient. A total of 44 CXHO active compounds and 820 targets were obtained after deleting duplicate targets, as shown in Table 1 and Figure 2. The Gene Cards database (https://www.genecards.org) were used collect GC related targets. GC as the keyword, species is human, Score ≥ 10 collected GC-related targets. Then, GC related targets and the targets of CXHO take intersection. The 480 common targets have discovered.

**Table 1:** The information of bioactive components of CXHO.

Herb name	Mol ID	Molecule name	OB (%)	DL
*Rhizoma Alpiniae Officinarum*	MOL001771	Poriferast-5-en-3beta-ol	36.91	0.75
	MOL002543	(2S, 3R)-2-(3, 4-Dimethoxyphenyl)-5, 7-dimethoxychroman-3-ol	51.89	0.37
	MOL002544	1, 7-Diphenyl-5-hydroxy-3-heptanone	61.9	0.18
	MOL002554	5-Methoxy-1, 7-diphenyl-3-heptanone	68.29	0.2
	MOL002556	7-Methoxy-8-(2′-ethoxy-3′-hydroxy-3′-methybutyl) coumarin	40.36	0.21
	MOL002563	Galangin	45.55	0.21
	MOL002565	Medicarpin	49.22	0.34
	MOL002575	Butyl-2-ethylhexyl phthalate	44.52	0.22
	MOL000354	Isorhamnetin	49.6	0.31
	MOL000358	β-Sitosterol	36.91	0.75
	MOL000359	Sitosterol	36.91	0.75
	MOL000422	Kaempferol	41.88	0.24
	MOL000098	Quercetin	46.43	0.28
*Piperis Longi Fructus*	MOL001555	ZINC03996196	52.35	0.62
	MOL001558	Sesamin	56.55	0.83
	MOL001559	Piperlonguminine	30.71	0.18
	MOL001560	Pipernonaline	51.32	0.41
	MOL001561	Dehydropipernonaline	47.73	0.41
	MOL001586	N-(2, 5-Dimethoxyphenyl)-4-methoxybenzamide	60.7	0.18
	MOL001588	N-Isobutyleicosa-2(E), 4(E), 8(Z)-trienamide	44.48	0.32
	MOL001589	N-Isobutyl-2, 4-icosadienamide	38.86	0.32
	MOL001592	Piperine	42.52	0.23
	MOL001594	Pisatin	88.05	0.64
	MOL001601	1, 2, 5, 6-Tetrahydrotanshinone	38.75	0.36
	MOL001607	ZINC03982454	36.91	0.76
	MOL001610	sylvatine	44	0.51
	MOL001614	(E, E, E)-11-(1, 3-Benzodioxol-5-yl)-N-(2-methylpropyl)-2, 4, 10-undecatrienenamide	42.72	0.43
	MOL001616	1-[1-Oxo-9(3, 4-methylenedioxyphenyl)-2E, 8E-nonadienyl] pyrrolidine	49.43	0.36
*Zingiber officinale Roseco*	MOL002464	1-Monolinolein	37.18	0.3
	MOL002501	[(1S)-3-[(E)-but-2-enyl]-2-methyl-4-oxo-1-cyclopent-2-enyl] (1R, 3R)-3-[(E)-3-methoxy-2-methyl-3-oxoprop-1-enyl]-2, 2-dimethylcyclopropane-1-carboxylate	62.52	0.31
	MOL002514	Sexangular tin	62.86	0.3
	MOL000358	β-Sitosterol	36.91	0.75
	MOL000359	Sitosterol	36.91	0.75
*Piper nigrum L*	MOL001589	N-Isobutyl-2, 4-icosadienamide	38.86	0.32
	MOL002840	Cryptopimaric acid	39.58	0.28
	MOL002843	(2E, 4E)-N-isobutyloctadeca-2, 4-dienamide	40.71	0.25
	MOL002847	Piperolein B	32.26	0.41
	MOL002848	cis-Piplartine	96.65	0.24
	MOL002857	55038-30-7	42.64	0.53
	MOL002862	Pipercide	42.72	0.43
	MOL002863	(E)-7-(1, 3-Benzodioxol-5-yl)-1-piperidino-hept-6-en-1-one	54.19	0.31
	MOL002864	(2E, 4Z)-5-(1, 3-Benzodioxol-5-yl)-1-piperidino-penta-2, 4-dien-1-one	37.52	0.23
	MOL002865	Trichostachine	63.63	0.2
	MOL002866	(2E, 4E, 8E)-9-(1, 3-Benzodioxol-5-yl)-N-isobutylnona-2, 4, 8-trienamide	65.9	0.33
	MOL000483	(Z)-3-(4-Hydroxy-3-methoxy-phenyl)-N-[2-(4-hydroxyphenyl) ethyl] acrylamide	118.35	0.26

DL = drug likeness, OB = oral bioavailability.

### 3.3. Identification of differentially expressed genes between GC and control tissue

**Figure 3.**
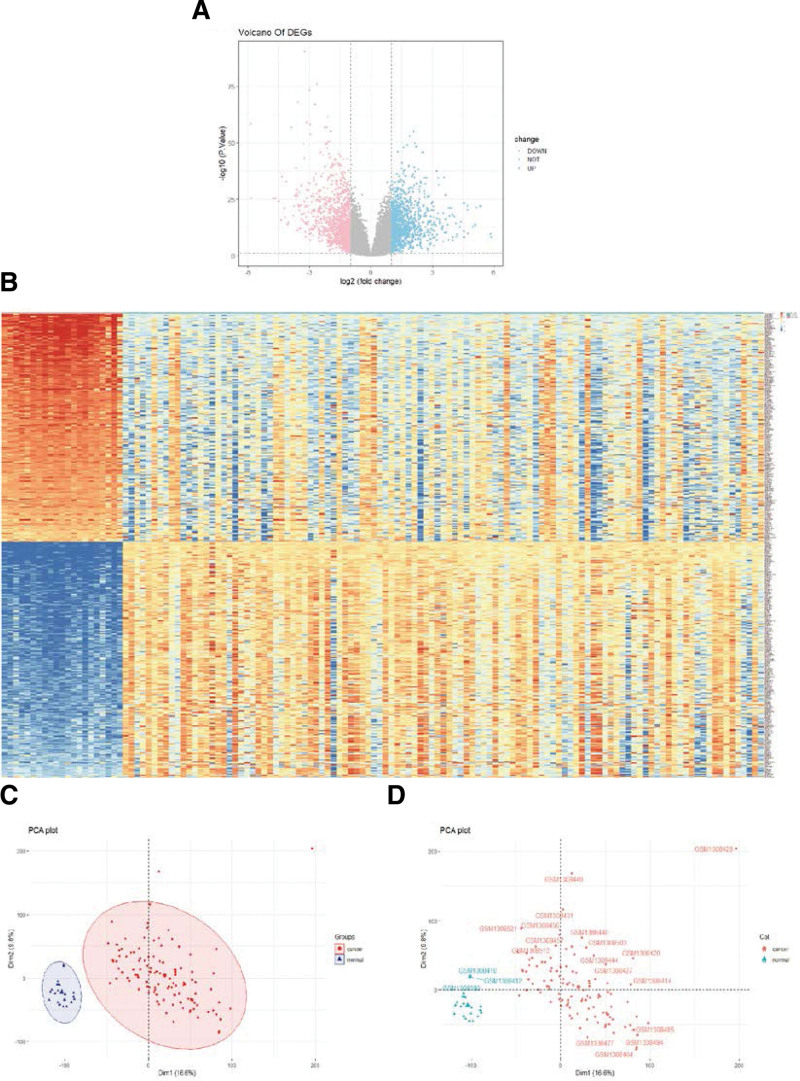
Differential gene analysis.

To identify candidate pivotal genes related to the progression of GC, we performed differential expression analysis between GC and control samples and obtained 462 DEGs, including 227 upregulated genes and 235 downregulated genes (*P* < .05 and |log_2_ FC| > 2; Fig. 3A). A heatmap showed a distinct expression pattern between the GC patients and the healthy controls (Fig. 3B). Principal component analysis further demonstrated a distinct expression mode between the tumoral and normal tissues (Fig. 3C and D).

### 3.4. Determination of CXHO-related hub genes in GC

**Figure 4.**
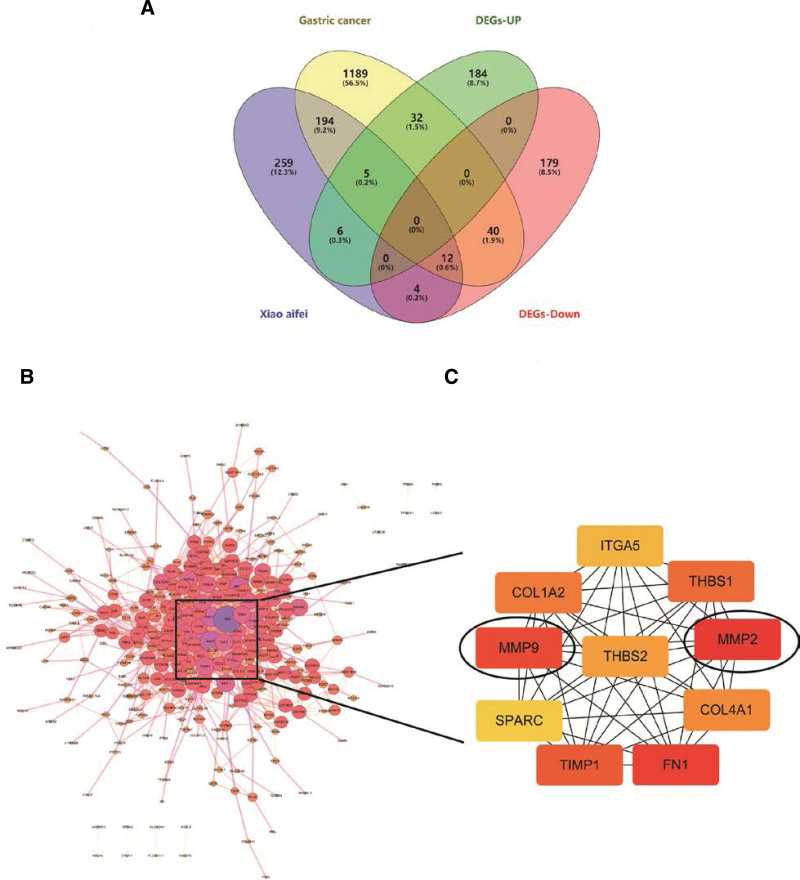
(A) Ween diagram between CXHO, gastric cancer, and DEGs. (B) The PPI network of DEGs, the larger the circle indicates that the gene interacts more with other proteins; (C) Top10 Hub genes, the darker the color, the higher the Degree value of this gene. CXHO = compound xiao-ai-fei honey ointment, DEGs = differentially expressed genes, PPI = protein–protein interaction.

To obtain the relevant target genes of CXHO in GC, we intersected the DEGs with target genes of CXHO and obtained 17 overlapping genes, namely, CCNA1, EPHX1, CYP1B1, MYLK, discoidin domain receptor 2 (DDR2), TGFB1, MMP2, FAP, CA9, SHH, CYP2C19, AKR1C3, PLA2G2A, MMP9, PKM, STS, and prostaglandin synthase COX-2 (PTGS2) (Fig. 4A). Meanwhile, we imported 462 DEGs into the STRING database resource to construct a PPI network (Fig. 4B and Table 2). We then further analyzed the PPI network using the Cytohubba plugin to screen the top 10 hub genes in the network, including ITGA5, THBS1, MMP2, COL4A1, FN1, TIMP1, SPARC, MMP9, THBS2, and COL1A2 (Fig. 4C). Notably, among these 10 hub genes, there were 2 shared genes among the 17 overlapping genes, i.e., MMP2 and MMP9. Thus, we mainly focused on these 2 genes in the downstream analysis.

**Table 2:** The information of 44 active compounds.

Name	Chemical name	Betweenness centrality	Closeness centrality	Topological coefficient	Degree
GLJ11	Isorhamnetin	0.17841789	0.44435548	0.10124654	190
GLJ10	Medicarpin	0.15251251	0.44152745	0.11428982	188
A	β-Sitosterol	0.11108302	0.39278132	0.12432432	164
GLJ9	Quercetin	0.08886846	0.41573034	0.13352381	150
GLJ7	Kaempferol	0.13702427	0.40540541	0.1336675	133
GLJ6	Galangin	0.06708438	0.40072202	0.15043605	129
GLJ2	(2S, 3R)-2-(3, 4-Dimethoxyphenyl)-5, 7-dimethoxychroman-3-ol	0.10133965	0.39445629	0.13394974	114
GLJ8	Butyl-2-ethylhexyl phthalate	0.098842	0.38461538	0.12507427	99
B	Sitosterol	0.01902873	0.35737283	0.14835165	98
GLJ4	5-Methoxy-1, 7-diphenyl-3-heptanone	0.09534998	0.38355218	0.1248538	95
HHJ1	Cryptopimaric acid	0.04532886	0.35875889	0.11901596	72
HHJ9	Piperlonguminine	0.06615594	0.36926148	0.11515152	71
BB3	Trichostachine	0.07080727	0.37273338	0.11191473	71
HHJ4	cis-Piplartine	0.05938577	0.3697535	0.12848233	65
GJ3	Sexangularetin	0.01674096	0.35968892	0.21115288	57
GLJ1	Poriferast-5-en-3beta-ol	0.01964964	0.35691318	0.15056022	51
BB8	Pisatin	0.03586387	0.35829567	0.12358804	43
GLJ3	1, 7-Diphenyl-5-hydroxy-3-heptanone	0.0107964	0.34839925	0.21283784	37
C	N-Isobutyl-2, 4-icosadienamide	0.01644353	0.35104364	0.10699588	40
HHJ8	(2E, 4Z)-5-(1, 3-Benzodioxol-5-yl)-1-piperidino-penta-2, 4-dien-1-one	0.01524692	0.35463259	0.22337043	34
GLJ5	7-Methoxy-8-(2′-ethoxy-3′-hydroxy-3′-methybutyl) coumarin	0.00469745	0.34883721	0.26194853	34
HHJ6	Pipercide	0.00796393	0.34796238	0.2540107	22
BB7	Piperine	0.01088405	0.34622583	0.27005348	22
HHJ3	Piperolein B	0.00494179	0.3428042	0.3	21
HHJ2	(2E, 4E)-N-isobutyloctadeca-2, 4-dienamide	0.02021358	0.3432282	0.12345679	21
HHJ10	(2E, 4E, 8E)-9-(1, 3-Benzodioxol-5-yl)-N-isobutylnona-2, 4, 8-trienamide	0.0043121	0.34111862	0.31785714	20
HHJ7	(E)-7-(1, 3-Benzodioxol-5-yl)-1-piperidino-hept-6-en-1-one	0.00465129	0.34238125	0.29666667	20
BB12	(E, E, E)-11-(1, 3-Benzodioxol-5-yl)-N-(2-methylpropyl)-2, 4, 10-undecatrienenamide	0.00427574	0.33293341	0.1962963	18
HHJ11	(Z)-3-(4-Hydroxy-3-methoxy-phenyl)-N-[2-(4-hydroxyphenyl) ethyl] acrylamide	0.00244413	0.34069982	0.32258065	15
BB1	ZINC03996196	0.00128913	0.34195933	0.29960317	14
HHJ5	55038-30-7	0.00315226	0.33820841	0.2974359	13
BB5	Pipernonaline	0.00227011	0.33862111	0.40104167	12
BB4	Dehydropipernonaline	0.00245669	0.33986528	0.35606061	12
BB2	Sesamin	0.00132999	0.34111862	0.23011364	11
BB13	1-[1-Oxo-9(3, 4-methylenedioxyphenyl)-2E, 8E-nonadienyl] pyrrolidine	0.00111816	0.33213645	0.40740741	9
BB11	Sylvatine	0.00391336	0.3051127	0.30519481	7
GJ2	(2S, 3R)-2-(3, 4-Dimethoxyphenyl)-5, 7-dimethoxychroman-3-ol	0.00020907	0.29318542	0.40625	6
GJ1	N-(2, 5-Dimethoxyphenyl)-4-methoxybenzamide	0.00023628	0.25970987	0.28	5
BB10	1, 2, 5, 6-Tetrahydrotanshinone	0.00020834	0.30212303	0.44	5
BB9	(2E, 4E, 8E)-9-(1, 3-Benzodioxol-5-yl)-N-isobutylnona-2, 4, 8-trienamide	0.00412356	0.31022918	0.23333333	5
BB6	1-Monolinolein	0.00036569	0.31022918	0.25833333	5

### 3.5. GO and KEGG pathway enrichment analysis

**Figure 5.**
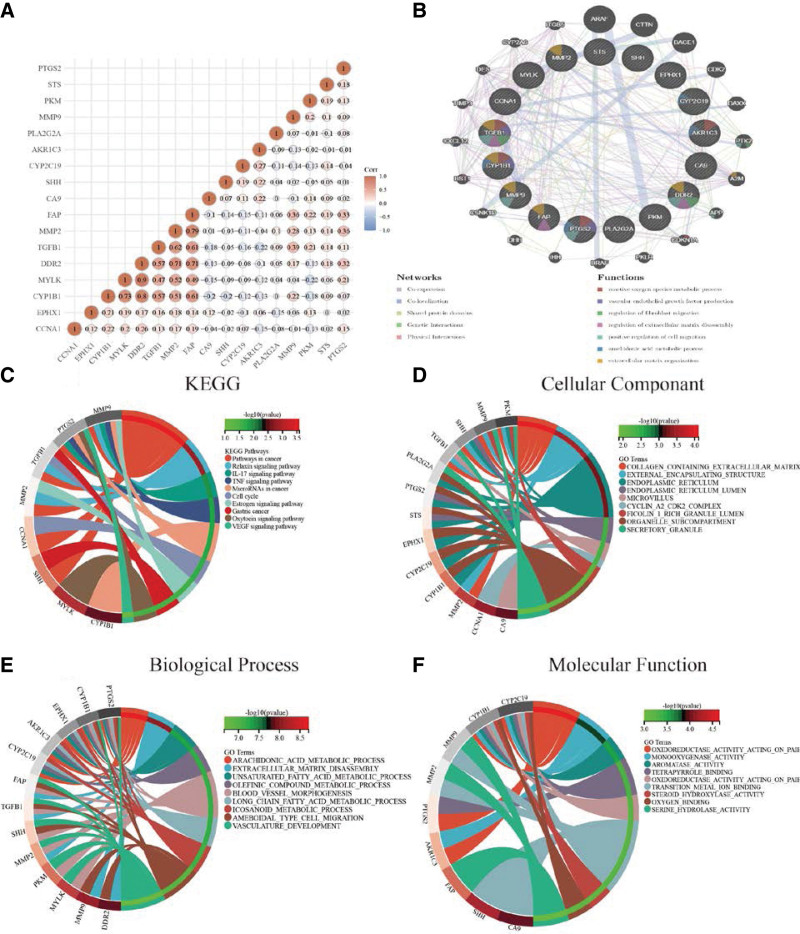
(A) Mainly focuses on the *P*-value, that is the correlation coefficient, if *P* < .05 believes that there is significantly, the correlation coefficient values range [‐1, 1], the negative number represents the negative correlation of the 2 gene expressions, and the positive value represents the positive correlation, the closer the value is to 1 or ‐1, the stronger the correlation between the two; the closer to 0, the weaker the correlation. (B) The size of the circle in the PPI network representing the degree to which the gene interacts with other proteins. (C–F) KEGG pathway, molecular functions, biological process, and cellular component enrichment maps of the overlapping genes. KEGG = Kyoto encyclopedia of genes and genomes.

To determine the target signaling pathways of CXHO in GC, we performed functional enrichment analysis for the 17 overlapping genes. First, we analyzed the relationship of the 17 CXHO-related genes and observed a strong positive correlation between CYP1B1, MYLK, DDR2, TGFB1, MMP2, and FAP, as well as a negative correlation between CA9, SHH, CYP2C19, and AKR1C3 (Fig. 5A). In addition, we found that the 17 overlapping genes were mainly enriched in reactive oxygen species metabolic process, vascular endothelial growth factor (VEGF) production, regulation of fibroblast migration, regulation of extracellular matrix disassembly, positive regulation of cell migration, arachidonic acid metabolic process, and extracellular matrix organization (Fig. 5B). The top 10 related KEGG signaling pathways consisted of pathways in cancer, relaxin signaling pathway, IL-17 signaling pathway, TNF signaling pathway, GC, cell cycle, microRNAs in cancer, etc (Fig. 5C). Enriched GO terms included olefinic compound metabolic process, steroid metabolic process, positive regulation of cell death, positive regulation of smooth muscle cell proliferation, etc. (Fig. 5D–F).

### 3.6. Verification of the prognostic value of 17 CXHO-related hub genes

**Figure 6.**
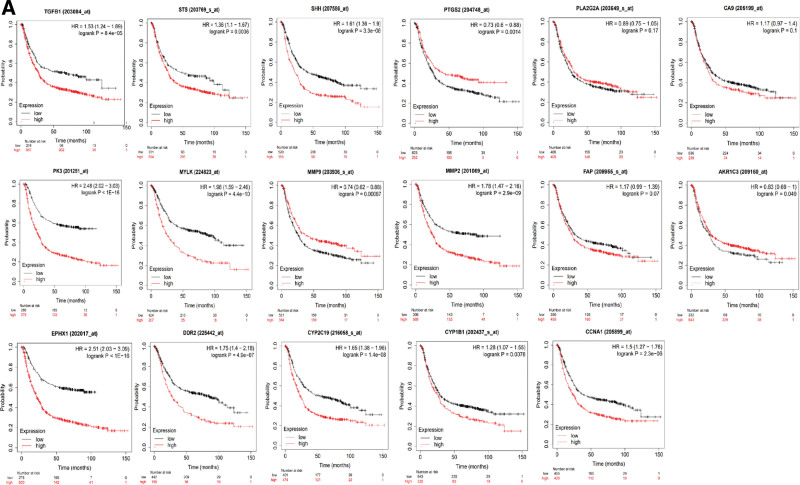
Kaplan–Meier survival analysis, and *P* < .05 was considered to have a significant impact on patient survival.

To validate the potential relevance of 17 CXHO-related hub genes, we next sought to investigate their prognostic capacity using 2 statistical approaches: Kaplan–Meier curve. Kaplan–Meier curves showed that the expression levels of CCNA1, EPHX1, CYP1B1, MYLK, DDR2, TGFB1, MMP2, SHH, CYP2C19, AKR1C3, MMP9, PKM, STS, and PTGS2 were significantly related to the survival of GC patients (Fig. 6), indicating the prognostic ability of these CXHO-related hub genes in GC.

### 3.7. Validation of the mRNA and protein levels of the 2 CXHO-related hub genes

**Figure 7.**
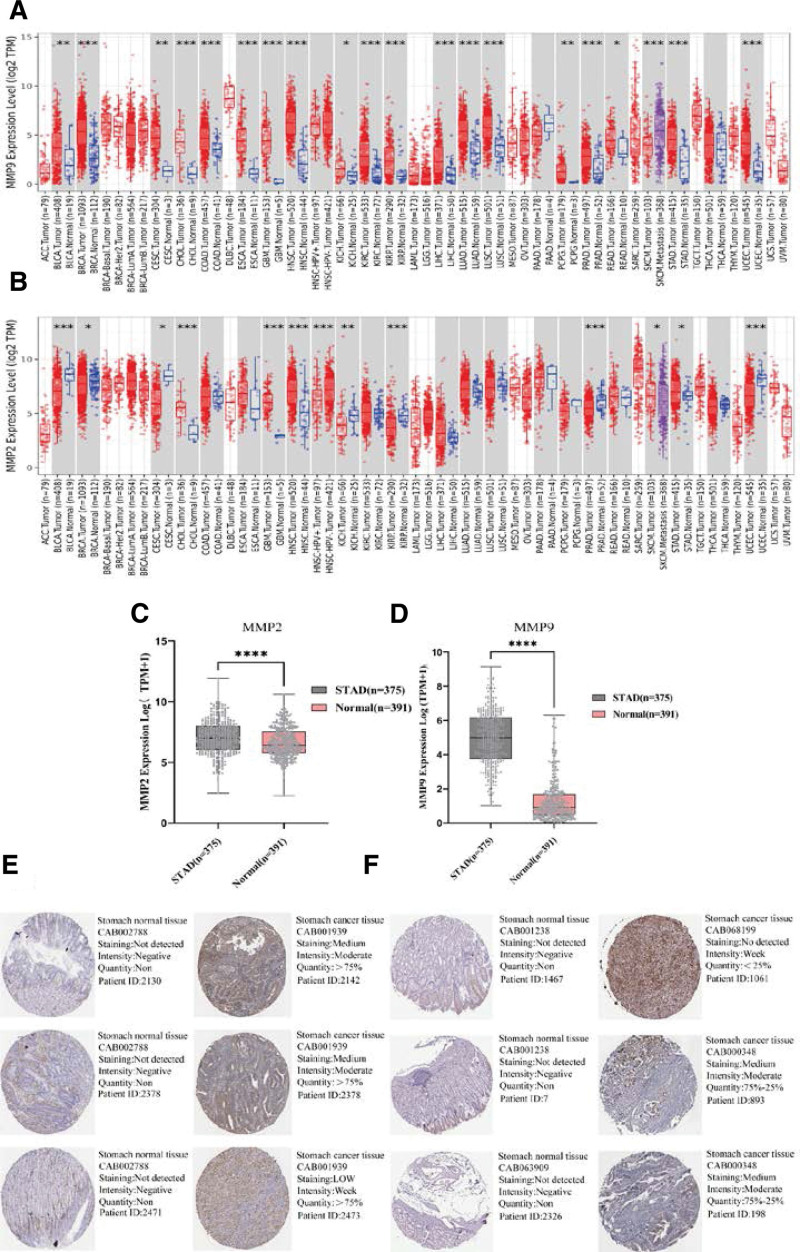
The expression level of MMP2, MMP9 in tumor tissues and normal tissues (A and B). *Represents *P* < .05. (C and D) The expression level of MMP9 and MMP2 in gastric cancer (n = 408) and normal tissues (n = 211) from the TCGA database; (E and F) gastric cancer and normal tissue immunohistochemical staining results.

To validate the expression of 2 CXHO-related hub genes, we employed 2 online bioinformatics tools to investigate their mRNA levels and utilized the Human Protein Atlas database to interrogate their protein levels. Pan-cancer analysis showed that MM9 was almost always downregulated among the 16 tumors, including GC; MMP2 was upregulated in BLCA, CESC, KICH, and UCEC and downregulated in CHOL, GBM, HNSC, KIRP, PRAD, SKCM, and GC (Fig. 7A and B). Concordantly, the findings based on the GEPEIA2 database demonstrated that the mRNA levels of MMP2 and MMP9 were upregulated in GC samples compared with normal samples (Fig. 7C and D). Immunohistochemical staining of MMP9 and MMP2 indicated that MMP2 and MMP9 were overexpressed in cancerous tissue compared with normal tissue (Fig. 7E and F).

### 3.8. Pan-cancer analysis of the relationship between the 2 CXHO-related hub genes and diverse immune subtypes

**Figure 8.**
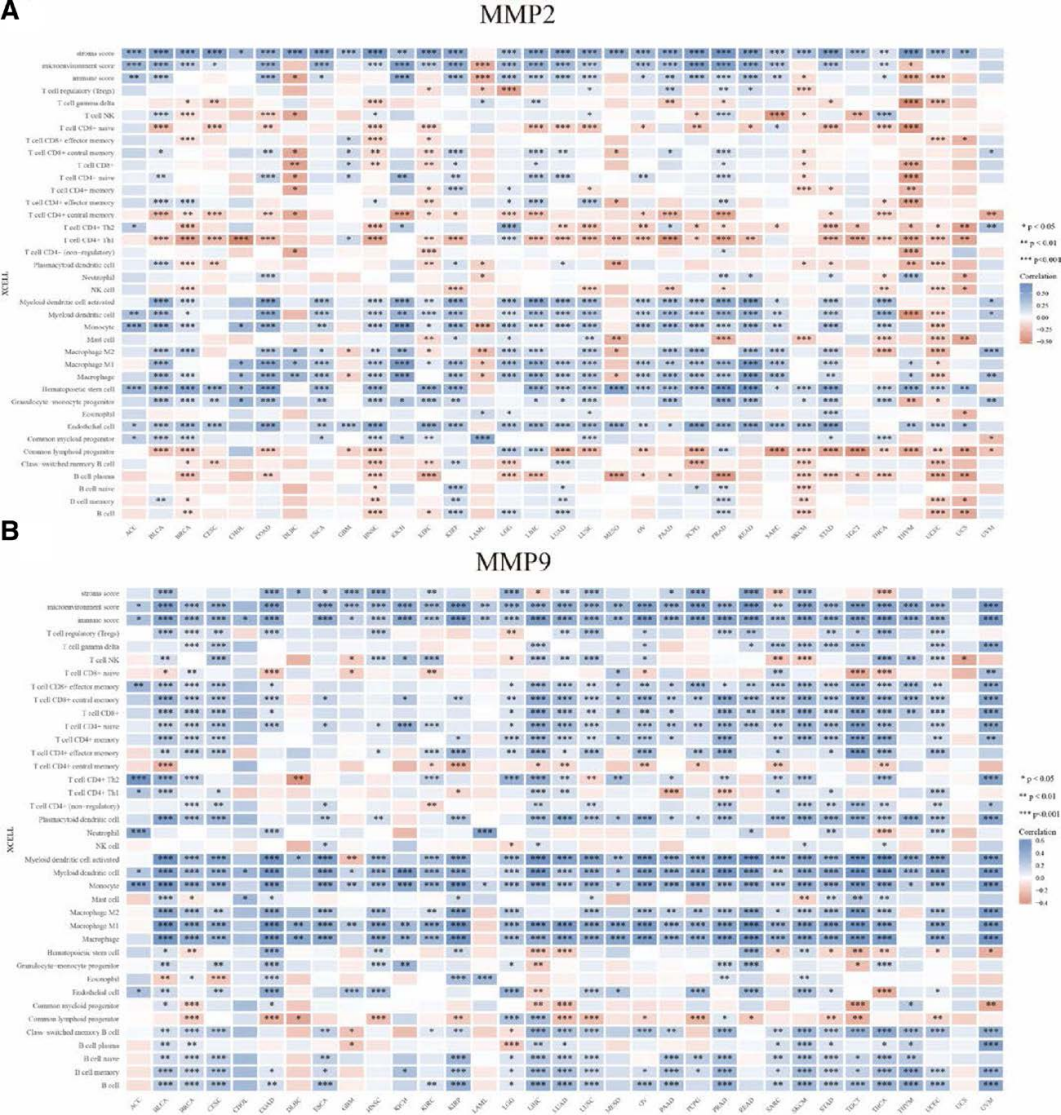
(A and B) Immune correlation of MMP2, MMP9: gastric cancer immune invasiveness score and MMP2, MMP9 gene expression spearman correlation analysis heat map in multiple tumor tissues, where abscissa represents different tumor tissues, ordinate represents different immune invasion scores, different colors represent correlation coefficients, negative values represent negative correlation, positive values represent positive correlation, the stronger the correlation, the darker the color, *P < .05, **P < .01, ***P < .001, asterisk represents the degree of importance (*P); the significance of both sets of samples passed the Wilcoxon test.

To further assess the relationship between hub genes and immune infiltrates, we employed the immunedeconv package in R to estimate the fraction of different immune cells using 6 independent algorithms, including TIMER, X Cell, MCP-counter, CIBERSORT, EPIC, and quantitative sequences. Correlation analysis was performed and showed that MMP2 was negatively correlated with B-cell plasma, CD4 + Th1/Th2 T cells, and naïve CD8 + T cells and positively correlated with endothelial cells, hematopoietic stem cells, monocytes, macrophages, and M1/M2 macrophages (Fig. 8A). On the other hand, MMP9 was inversely correlated with the common lymphoid progenitor hematopoietic stem cell and positively correlated with B-cell and B-cell memory, B-cell naïve, T-cell CD4 + nonregulatory, T-cell CD4 + memory, T-cell CD4 + naïve, T-cell CD8+, T-cell CD8 + central memory, T-cell CD8 + effector, and Treg cells (Fig. 8B). Remarkably, monocytes, macrophages, and M1/M2 macrophages were positively correlated with MMP2 and MMP9, indicating a key role in immune escape and immunosuppression in the development of GC.

### 3.9. Molecular docking analysis

**Figure 9.**
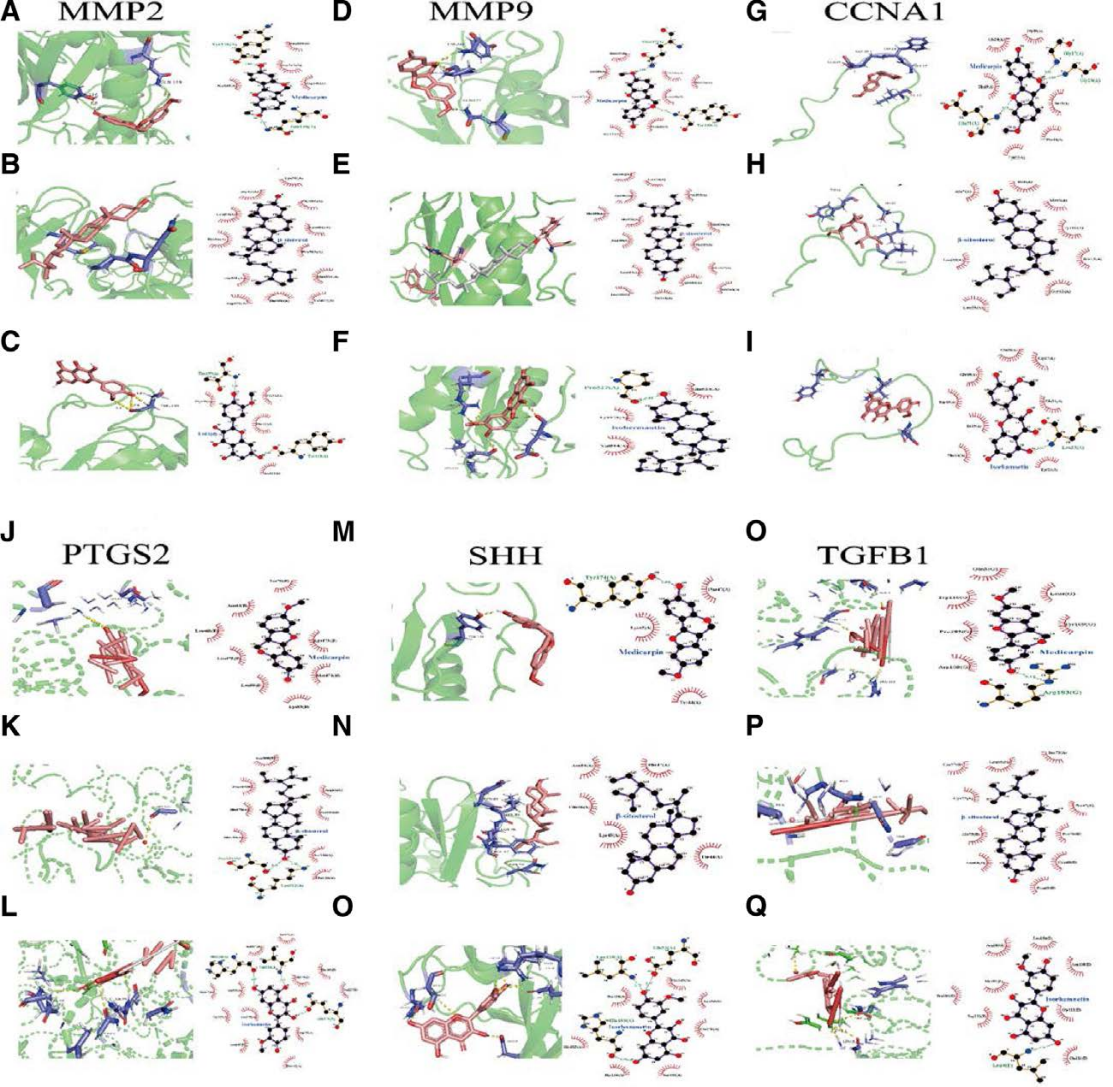
Molecular binding models of the Hub targets with bioactive ingredients. (A) MMP2 with medicarpin, affinity = ‐4.48 kcal/mol; (B) MMP2 with β-sitosterol, affinity = ‐6.03 kcal/mol; (C) MMP2 with isorhamnetin, affinity = ‐3.8 kcal/mol; (D) MMP9 with medicarpin, affinity = ‐5.92 kcal/mol; (E) MMP9 with β-sitosterol, affinity = ‐9.08 kcal/mol; (F) MMP9 with isorhamnetin, affinity = ‐5.6 kcal/mol; (G) CCNA1 with medicarpin, affinity = ‐6.27 kcal/mol; (H) CCNA1 with β-sitosterol, affinity = ‐5.22 kcal/mol; (I) CCNA1 with isorhamnetin, affinity = 5.27 kcal/mol; (J) PTGS2 with medicarpin, affinity = ‐5.01 kcal/mol; (K) PTGS2 with β-sitosterol, affinity = ‐6.36 kcal/mol; (L) PTGS2 with isorhamnetin, affinity = ‐4.96 kcal/mol; (M) SHH with medicarpin, affinity = ‐5.55 kcal/mol; (N) SHH with β-sitosterol, affinity = ‐6.15 kcal/mol; (O) SHH with isorhamnetin, affinity = ‐4.9 kcal/mol; (P) TGFB1 with Medicarpin, affinity = ‐5.3 kcal/mol; (Q) TGFB1 with β-sitosterol, affinity = ‐4.92 kcal/mol; (R) TGFB1 with isorhamnetin, affinity = ‐3.9 kcal/mol.

To further confirm the efficacy of CXHO in treating GC, we selected 3 compounds with the highest degree values in CXHO for molecular docking, namely SIT, IH, and medicarpin, as they were found to have more genes in common with the 17 core genes (see Fig. S1, Supplemental Digital Content, http://links.lww.com/MD/J419, which demonstrates MMP2, PTGS2, CCNA1, SHH, TGFB1, and MMP9 were identified as the common genes of these 3 active compounds). Our findings revealed that SIT, IH, and medicarpin demonstrated a strong binding affinity to MMP2, PTGS2, CCNA1, SHH, TGFB1, and MMP9, as depicted in Figure 9 and Table 3.

**Table 3:** Docking scores of targets with components (kcal·mol^−1^).

Target name	PDBID	Medicarpin	Components β-sitosterol	Isorhamnetin
MMP9	6ESM	‐5.92	‐9.08	‐5.6
MMP2	1CK7	‐4.48	‐6.3	‐3.8
CCNA1	Q9UNG8	‐6.27	‐5.22	‐5.27
PTGS2	5F19	‐5.01	‐6.36	‐4.96
SHH	6PJV	‐5.55	‐6.15	‐4.9
TGFB1	4KV5	‐5.3	‐4.92	‐3.9

### 3.10. Experimental validation

**Figure 10.**
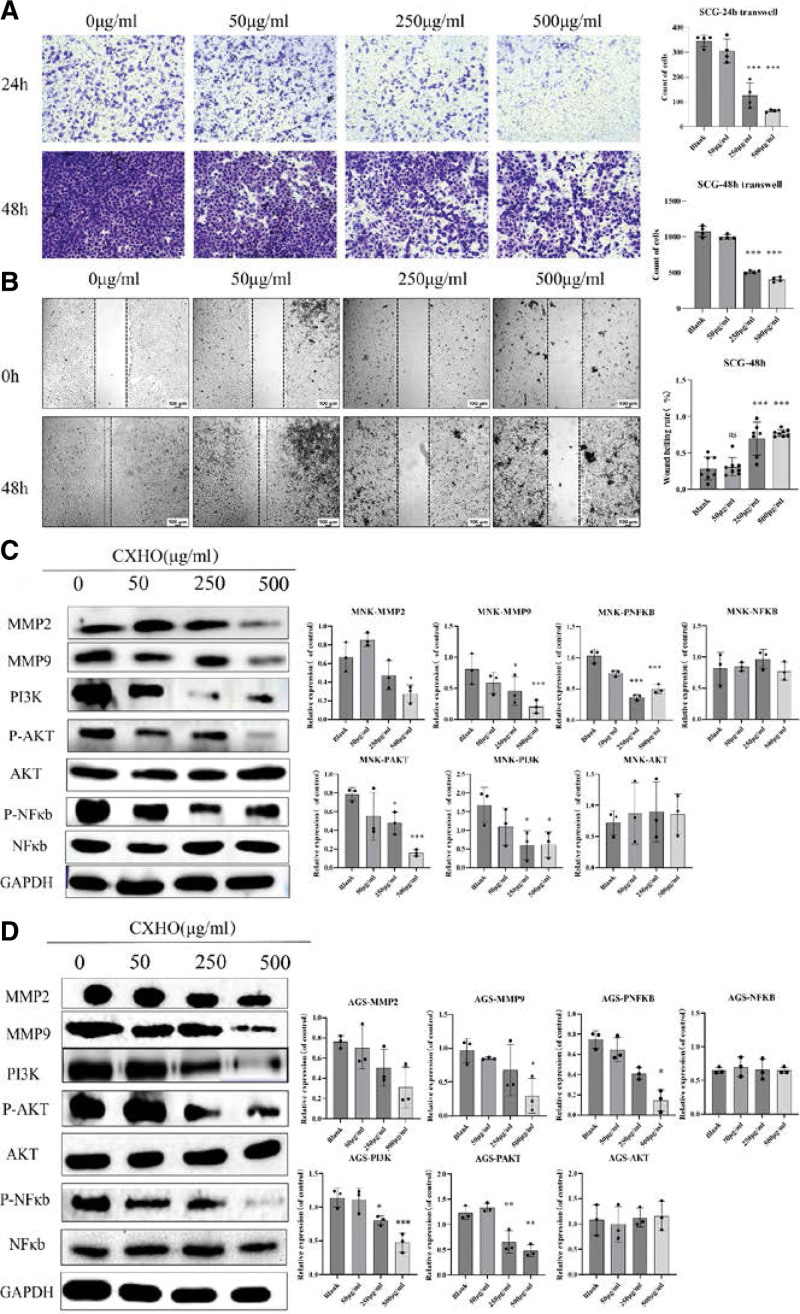
(A) The cell migration of AGS cells detected by Transwell chamber assay; (B) the cell migration of AGS cells detected by wound healing assay; the expression level of MMP2, MMP2, PI3K/AKT, NF-κb after treated with CXHO was detected by western blot in AGS cells (C) and MNK-45 cells (D). CXHO = compound xiao-ai-fei honey ointment.

To verify the effects of CXHO on GC cell migration, AGS cells were selected for CXHO treatment for 24 hours and 48 hours, the results showed that CXHO can reduce migration of GC cells by dose-dependent characteristics, as shown in (Fig. 10A and B). In summary, these data confirmed that CXHO may inhibit the metastasis of GC. We further validated the regulation of CXHO on the expression of potential anti-tumor targets identified by network pharmacology. Pretreatment of AGS cells and MNK-45 cells with CXHO (50, 250, and 500 μg/mL) resulted in significant inhibition and upregulation of PI3K, AKT, and NFKB, MMP9, and MMP2. As shown in Figure 10C and D. These results confirmed that CXHO may regulate cell migration through the MMP9/MMP2-PI3K-NF-κB pathway.

## 4. Discussion

GC is one of the most frequent malignancies, with high morbidity and mortality worldwide.^[35]^ The early symptoms of GC are not obvious, leading to a low early diagnosis rate.^[2]^ At present, the treatment of GC is mainly based on combination therapy strategies, including surgical operation,^[36]^ radiotherapy, chemotherapy,^[37]^ molecular targeted therapy,^[38]^ and immunotherapy.^[39]^ However, these treatments have limited efficacy due to drug resistance. Therefore, it is necessary to find novel drugs to improve the efficacy of current combination therapy. TCM has a long history, and many TCM are reported to have anticancer effects.^[40,41]^

In this study, we found that CXHO contains a total of 45 bioactive compounds, and these ingredients were identified to target 480 genes in GC. Among these, 17 genes were further determined as the core targets of CXHO against GC, including CCNA1, EPHX1, CYP1B1, MYLK, DDR2, TGFB1, MMP2, FAP, CA9, SHH, CYP2C19, AKR1C3, PLA2G2A, MMP9, PKM, STS, and PTGS2. Functional enrichment analysis demonstrated that a variety of signaling pathways were associated with CXHO, such as ovarian steroidogenesis and proteoglycans in cancer. Survival analysis showed that the expression levels of CCNA1, EPHX1, CYP1B1, MYLK, DDR2, TGFB1, MMP2, SHH, CYP2C19, AKR1C3, MMP9, PKM, STS, and PTGS2 were significantly related to the prognostic outcomes of GC patients. Finally, MMP2 and MMP9 were identified and validated as hub genes in GC. In addition, molecular docking analysis confirmed that MMP2, MMP9, CCNA1, TGFB1, PTGS2, and SHH had strong binding affinities with most bioactive compounds SIT, IH, and medicarpin.

IH was reported to have anti-inflammatory and antiproliferation activity in many cancers^[42–45]^ and induce a cytotoxic effect by inhibiting ROS in tumor cells.^[46]^ Medicarpin is a natural phytoestrogen usually found in extracts of various legumes,^[47]^ and can exert anti-osteoclast and anti-inflammatory activity^[48]^ as well as proapoptotic properties in leukemia cells.^[49,50]^ SIT is one of the most plentiful natural plant sterols^[51]^ and is involved in the activation of the tumor suppressor protein P53. Several studies have reported that SIT can induce anticancer effects through several different mechanisms. Ein et al^[52]^ confirmed that SIT could affect the growth and apoptosis of tumor cells by increasing the phosphorylation level of AMPK, which is associated with cancer malignancy.^[53,54]^ According to Nuraniye Rahman report in 2008,^[4]^ CXHO extracts can impede the proliferation of BGC-823 GC cells. Meanwhile, Mirensha Yakufu^[8,9]^ conducted a study on the effect of CXHO ethanol extract on tumor growth in MCF mice. The results revealed that CXHO ethanol extract’s inhibitory rate was comparable to that of the positive drug 5-Fu, while the mice’s weight did not decrease significantly. This suggests that CXHO can hinder tumor growth without causing significant toxicity to the mice.

The potential key genes related to CXHO all have their own relationship with the tumor. CYP1B1 is a member of the cytochrome P450 family, it is helping cancer cells resisted the toxicity of chemical drugs by oxidation and metabolism of many anticancer drugs. In addition, CYP1B1 caused DNA single-strand breakage and DNA destruction. Thus, CYP1B1 have potential carcinogenic effects.^[55,56]^ DDR2 belongs to the receptor tyrosine kinases subfamily. Wang YG^[57]^ found DDR2 was higher expressed in GC tissues and cells. In xenograft models, DDR2 high expression induced Tumorigenesis, influence GC cells migration and promoted cell invasion through AKT phosphorylation and mTORC2 activation. TGF-β1 play dual role in GC, not only TGF-β1 was promoted tumorigenesis, suppressing immunesurveillance and increase epithelial-mesenchymal transition, but also, TGF-β1 inhibits tumor development by directly suppressed cell cycle, caused growth arrest and activated apoptotic pathways.^[58,59]^ FAP has been revealed have pro-tumorigenic activity, both through enzymatic and nonenzymatic ways.^[60,61]^ The adaptation of cancer cells to hypoxia and acidosis is a key process in cancer progression.^[62]^ CA9 is induced hypoxia by the HIF-1α pathway,^[63]^ it has played an important role in tumor acidification and hydration of carbon dioxide.^[64]^ CA9 is high expressed in various cancers and usually not expressed in normal tissues.^[65]^ CA9 be regarded as a marker of hypoxia. Moreover, previous reports have suggested that overexpression of CA9 and HIF-1α is related with a poor prognosis of GC.^[66–68]^ SHH/GLI signaling is closely related to embryonic development and tissue homeostasis.^[69–71]^ Recent studies have suggested that the imbalance of this signal axis could contribute to tumor metastasis and drug resistance. In addition, SHH signaling impact cancer progression by regulated cell proliferation, CSCs amplification and tumor malignant metastasis.^[72,73]^ Pyruvate kinase isoform M2 (PKM2) is one of the isoenzymes of pyruvate kinase and is a key glycolytic enzyme. Abnormal PKM2 expression promotes malignant tumor metastasis and directly associated with clinical progression of the digestive system solid tumors.^[74,75]^ In addition, malignant proliferation and poor prognosis of GC also directly associated with PKM2 expression.^[76,77]^ Smyth EM^[78]^ revealed that knock down PKM2 has suppressing G1-S phase transition of GC cells, particularly attenuates cell migration in vivo and vitro, increased autophagy, that is could contributed by mediating PI3K-Akt signaling pathway. PTGS2 belong the family of lipid mediators and responsible for the conversion of arachidonic acid into various prostaglandins.^[79,80]^ Uefuji K^[81]^ found that COX-2 overexpression was related with increased PGE2 biosynthesis and angiogenesis in GC. In addition, COX-2 could increase cancer cells produce VEGF and TGF-β, thereby promoting endothelial cell migration. Moreover, VEGF was an independent prognostic factor for GC prognosis.^[82]^ Cheng J^[83]^ confirmed that COX-2 is involved in the immunosuppression of GC may involve PGE2 induce dendritic cells in the tumor microenvironment (TME) to lose function, which cannot effectively present tumor antigens, and ultimately T cells cannot recognize or kill cancer cells. Transforming growth factor-β (TGF-β) pathway is a fundamental signaling pathway that plays an important role in tissue homeostasis. With tumors malignant progression, the genome often accumulates mutations in the TGF-β receptor system, rendering cancer cells unresponsive to TGF-β.^[84]^ This converts TGF-β into tumor promoters, resulting in increased invasion and metastasis.^[85]^ MMP9 is secreted to the surface of cells by binding with the surface receptor CD44, and then proteolysis activates TGF-β.^[86]^ In addition, MMP2, MMP9, and MMP14 indirectly regulate the bioactivity of TGF-β by cutting off the potential TGF-β binding protein 1 (LTBP-1) of the ECM component, and dissolving the ECM-bound TGF-β.^[87,88]^

MMPs, which are a family of proteases, have been strongly associated with tumorigenesis. They regulate various physiological processes and mediate changes in the microenvironment during tumor progression.^[89]^ MMP9, in particular, plays a crucial role in tumor angiogenesis by regulating the VEGF, the most effective inducer and primary therapeutic target for tumor angiogenesis.^[90,91]^ Tumor growth cannot occur in MMP9-deficient mice. However, tumor growth can be restored by transplanting CD11b-positive bone marrow cells from MMP9-sufficiency mice, suggesting that MMP9 is necessary for tumor angiogenesis.^[92]^ Therefore, MMP9 could be an important target for adjuvant therapy.

**Figure 11.**
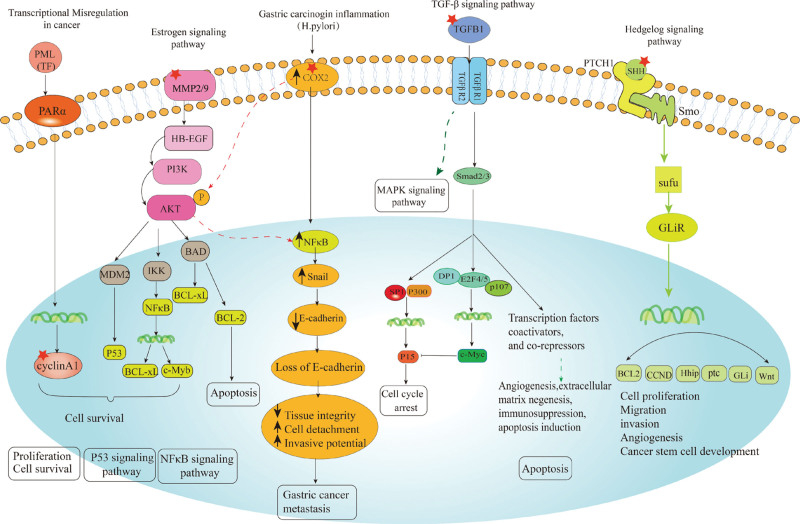
The molecular mechanism schematic of CXHO inhibited proliferation migration and induced apoptosis, cell cycle arrest by NF-κB, P53, estrogen signaling pathway, hedgehog signaling pathway, and TGF-β1 signaling pathway. The star represents the predicted key target of CXHO in related pathways. CXHO = compound xiao-ai-fei honey ointment, TGF-β1 = transforming growth factor-β1.

Tumor cells interact with various immune cells in the TME, leading to the development of malignant tumors. Among the immune cells, macrophages are the most abundant in the TME.^[93]^ Macrophages can be classified into M1 and M2 subsets, which produce different cytokines that regulate tumor immunity. M1 macrophages produce cytokines that induce anticancer responses, whereas M2 macrophages produce cytokines that stimulate the Th2 immune response and regulatory T cell activation, promote Th2 cell proliferation, and induce immune tolerance. In GC, M2 macrophages are associated with poor prognosis, and their polarization is promoted by GC-derived mesenchymal stromal cells. immune infiltration analysis showed that MMP9 and MMP2 are positively correlated with M1/M2 macrophages and their expression can be inhibited by CXHO, which can suppress macrophage differentiation, inhibit GC metastasis, and improve prognosis. In addition, we summarized several possible molecular mechanisms of CXHO influence on GC, as shown in Figure 11. Effective blocked MMP2, MMP9, and NF-κb in GC cells AGS and MNK-45 after CXHO pretreatment may contribute to its efficacy. CXHO affects the migration of GC cells, which may help inhibit GC metastasis. In summary, our results suggested that CXHO may inhibit STAD progression through PI3K-AKT-NF-ΚB and MMP2, MMP9 signaling pathway regulation Figure 11. In addition, the detailed pharmacological mechanisms by which CXHO improves STAD will be investigated in our future studies.

## 5. Conclusion

This study utilized network analysis and bioinformatics to investigate the potential of CXHO in treating GC by exploring its active components and putative targets. Through PPI and CT network analysis, 17 important targets were identified, and 2 pivotal target genes, MMP2 and MMP9, were validated as upregulated in GC and involved in the tumor immune microenvironment. Our findings suggest that CXHO, with its multiple components, targets, and pathways, shows promise as a potential drug for treating GC. Furthermore, molecular docking results indicated strong binding affinity between the 3 most active compounds, SIT, medicarpin, and IH, with CCNA1, SHH, MMP9, MMP2, TGFB1, and PTGS2. These results further validated the feasibility of CXHO in treating GC. All these findings support CXHO as a promising drug for the treatment of GC. Although CXHO has been used as Uyghur folk medicine to treat cancer in clinical for years, the current studies still lack biological experiments and further validation will be conducted to justify the hypothesis and conclusions.

## Acknowledgments

We are grateful to all the researchers who provided the data we used.

## Author contributions

**Conceptualization:** Kayisaier Abudurousuli.

**Data curation:** Sendaer Hailati, Meng Yuan Han.

**Formal analysis:** Ziruo Talihati, Jimilihan Simayi.

**Funding acquisition:** Wenting Zhou.

**Investigation:** Kayisaier Abudurousuli, Ziruo Talihati.

**Methodology:** Kayisaier Abudurousuli.

**Project administration:** Wenting Zhou.

**Resources:** Dilihuma Dilimulati, Nuerbiye Nueraihemaiti.

**Software:** Kayisaier Abudurousuli, Dilihuma Dilimulati, Nuerbiye Nueraihemaiti.

**Supervision:** Muhadaisi Nuer, Nawaz Khan.

**Validation:** Kayisaier Abudurousuli.

**Visualization:** Ziruo Talihati, Nulibiya Maihemuti.

**Writing – original draft:** Kayisaier Abudurousuli.

**Writing – review & editing:** Wenting Zhou.

## Supplementary Material


