# Supplementary material for: Investigation of target genes and potential mechanisms related to compound Xiao-ai-fei honey ointment based on network pharmacology and bioinformatics analysis
Source: Medicine (Baltimore). 2023 Aug 11;102(32):e34629. doi: 10.1097/MD.0000000000034629 (PMC10419591; doi:10.1097/MD.0000000000034629)
Supplement: Supplementary file 1 [file medi-102-e34629-s001.pptx]

## Slide 1
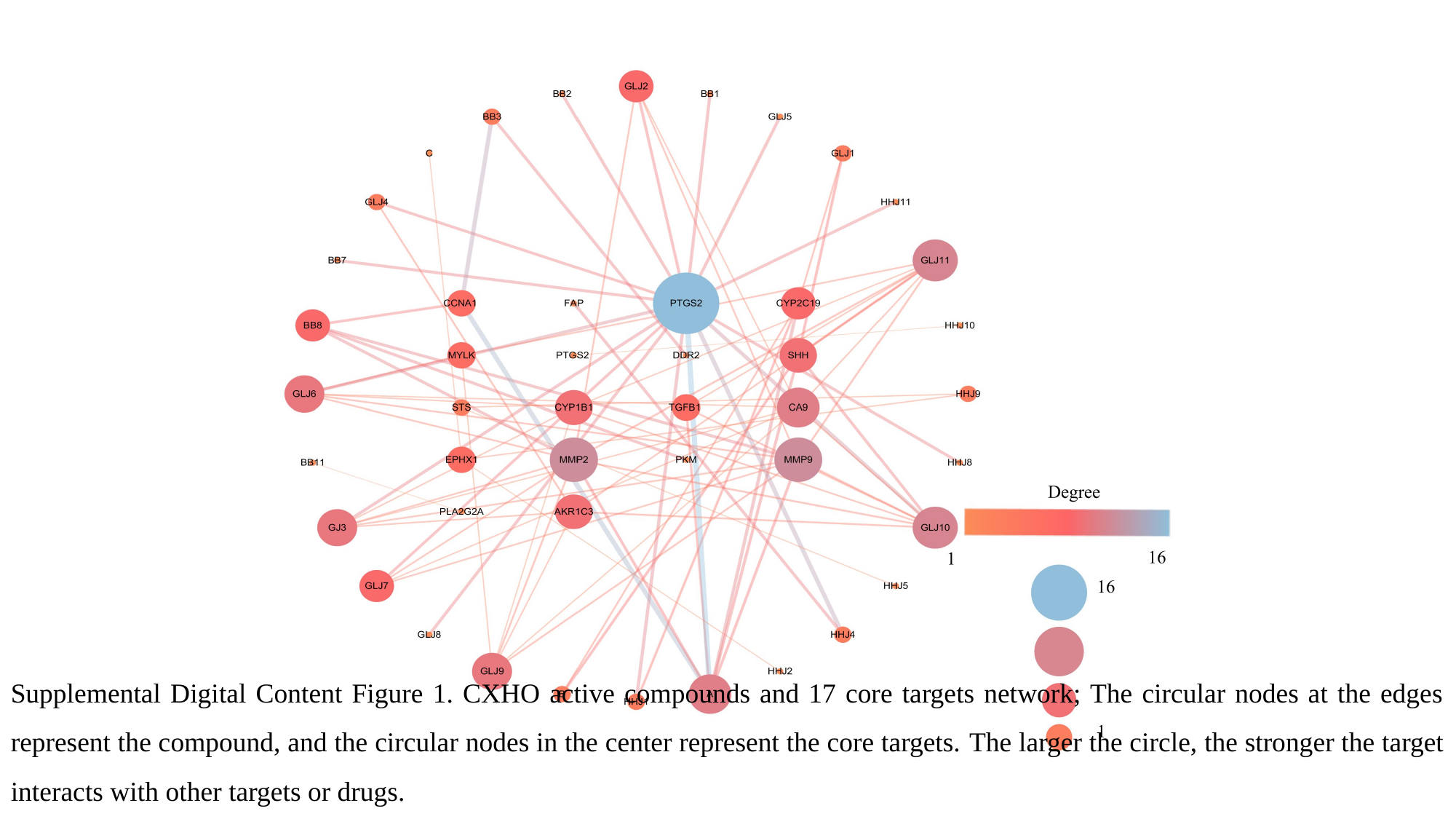

Supplemental Digital Content Figure 1. CXHO active compounds and 17 core targets network; The circular nodes at the edges represent the compound, and the circular nodes in the center represent the core targets. The larger the circle, the stronger the target interacts with other targets or drugs.
